# Safety and immunogenicity of a recombinant COVID-19 vaccine (Sf9 cells) in healthy population aged 18 years or older: two single-center, randomised, double-blind, placebo-controlled, phase 1 and phase 2 trials

**DOI:** 10.1038/s41392-021-00692-3

**Published:** 2021-07-15

**Authors:** Fan-Yue Meng, Fan Gao, Si-Yue Jia, Xiang-Hong Wu, Jing-Xin Li, Xi-Ling Guo, Jia-Lu Zhang, Bo-Pei Cui, Zhi-Ming Wu, Ming-Wei Wei, Zhi-Long Ma, Hai-Lin Peng, Hong-Xing Pan, Lin Fan, Jing Zhang, Jiu-Qin Wan, Zhong-Kui Zhu, Xue-Wen Wang, Feng-Cai Zhu

**Affiliations:** 1 NHC Key Laboratory of Enteric Pathogenic Microbiology, Jiangsu Provincial Center for Disease Control and Prevention, Nanjing, China; 2 China National Institute for Food and Drug Control, Beijing, China; 3 Sheyang County Center for Disease Control and Prevention, Yancheng, China; 4 Jiangdu District Center for Disease Control and Prevention, Yangzhou, China; 5 Taizhou Center for Disease Control and Prevention, Taizhou, China; 6 Jiangsu Taizhou People’s Hospital, Taizhou, China; 7 Shanghai Canming Medical Technology Co., Ltd, Shanghai, China

**Keywords:** Vaccines, Infectious diseases

## Abstract

COVID-19 vaccines from multiple manufacturers are needed to cope with the problem of insufficient supply. We did two single-center, randomised, double-blind, placebo-controlled phase 1 and phase 2 trials to assess the safety, tolerability and immunogenicity of a recombinant COVID-19 vaccine (Sf9 cells) in healthy population aged 18 years or older in China. Eligible participants were enrolled, the ratio of candidate vaccine and placebo within each dose group was 3:1 (phase 1) or 5:1 (phase 2). From August 28, 2020, 168 participants were sequentially enrolled and randomly assigned to receive the low dose vaccine, high dose vaccine or placebo with the schedule of 0, 28 days or 0, 14, 28 days in phase 1 trial. From November 18, 2020, 960 participants were randomly assigned to receive the low dose vaccine, high dose vaccine or placebo with the schedule of 0, 21 days or 0, 14, 28 days in phase 2 trial. The most common solicited injection site adverse reaction within 7 days in both trials was pain. The most common solicited systematic adverse reactions within 7 days were fatigue, cough, sore throat, fever and headache. ELISA antibodies and neutralising antibodies increased at 14 days, and peaked at 28 days (phase 1) or 30 days (phase 2) after the last dose vaccination. The GMTs of neutralising antibody against live SARS-CoV-2 at 28 days or 30 days after the last dose vaccination were highest in the adult high dose group (0, 14, 28 days), with 102.9 (95% CI 61.9–171.2) and 102.6 (95% CI 75.2–140.1) in phase 1 and phase 2 trials, respectively. Specific T-cell response peaked at 14 days after the last dose vaccination in phase 1 trial. This vaccine is safe, and induced significant immune responses after three doses of vaccination.

## Introduction

Following the outbreak of severe acute respiratory syndrome coronavirus (SARS-CoV) and middle east respiratory syndrome coronavirus (MERS-CoV), severe acute respiratory syndrome coronavirus 2 (SARS-CoV-2) is the third highly pathogenic coronavirus that has emerged in humans during the past 20 years. SARS-CoV-2 infection is accelerating globally, leading to an increase in morbidity and mortality. As of June 16 2021, there have been over 170 million confirmed COVID-19 cases, including nearly 4 million deaths, reported to World Health Organization (WHO).^1^

Nowadays, there are over 280 COVID-19 vaccines in development worldwide according to the survey of WHO.^2^ Among them, 102 vaccines have been approved for clinical trials, including protein subunit (*n* = 32, accounted for 31%), viral vector (*n* = 21, accounted for 21%), inactivated vaccines (*n* = 16, accounted for 16%), DNA or RNA vaccines (*n* = 26, accounted for 25%), VLP vaccines (*n* = 5, accounted for 5%), and live attenuated vaccines (*n* = 2, accounted for 2%). Up to now, 25 COVID-19 vaccines are in phase 3 clinical trials, and the ‘first wave’ of eight COVID-19 vaccines have proclaimed their preliminary efficacy results, including the mRNA vaccine, non-replicating viral vectored vaccine, protein subunit vaccine and inactivated vaccine, with the efficacy between 50% and 95%.^3–8^

Except the ‘first wave’ of COVID-19 vaccines, there are so many candidate vaccines underdevelopment in pipeline with their efficacy undetermined, particularly for vaccines from protein subunit technology platform has accounted for more than 30 percent. Besides, recombinant subunit vaccines target a wider range of population, which are safer for people with relatively low immunity, the elderly and children, and are easy for storage and logistics. Protein subunit vaccines are promising in the development of COVID-19 vaccines. Our recombinant COVID-19 vaccine uses baculovirus as a vector and expresses the RBD region of the SARS-CoV-2 spike protein receptor binding domain in Sf9 cells, which was isolated and purified and added adjuvant to prevent COVID-19.

Herein, we report the first analysis results at 28 days (phase 1) or 30 days (phase 2) after the full doses of vaccination to assess the safety and immunogenicity of a recombinant COVID-19 vaccine (Sf9 cells) in healthy population aged 18 years or older in China.

## Results

### Study participants

**Fig. 1:**
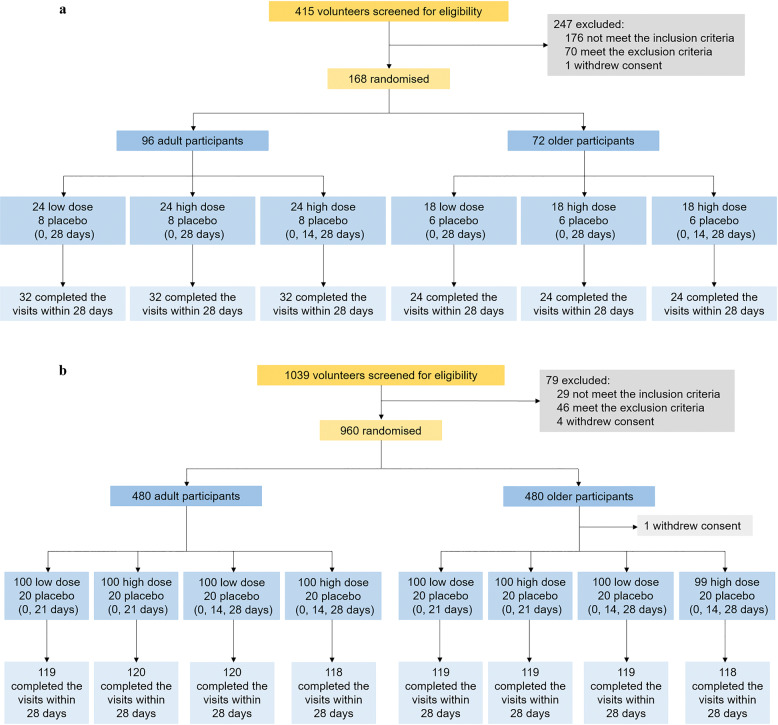
Trial profile. **a** In phase 1 trial, 415 volunteers were recruited and screened for eligibility, among which 168 participants were sequentially enrolled and randomly assigned. All participants completed the full doses of vaccination and the planned visits within 28 days after the last dose vaccination. **b** In phase 2 trial, 1039 volunteers were recruited and screened for eligibility, among which 960 participants were enrolled and randomly assigned. 959 participants received the first dose vaccination, with 1 participant refused to receive vaccination after randomization. 952 participants completed the full doses of vaccination and the planned visits within 30 days after the last dose vaccination

From August 28, 2020, 415 volunteers were recruited and screened for eligibility in phase 1 trial, among which 168 participants were sequentially enrolled and randomly assigned, including 96 adults (18–55 years) and 72 older individuals (56 years or older), to receive the low dose vaccine (0, 28 days, *n* = 42), high dose vaccine (0, 28 days, *n* = 42), high dose vaccine (0, 14, 28 days, *n* = 42) or placebo (0, 28 days or 0, 14, 28 days, *n* = 42) (Fig. 1).

From November 18, 2020, 1039 volunteers were recruited and screened for eligibility in phase 2 trial, among which 960 participants were enrolled and randomly assigned, including 480 adults (18–59 years) and 480 older individuals (60 years or older), to receive the low dose vaccine (0, 21 days, *n* = 200), high dose vaccine (0, 21 days, *n* = 200), low dose vaccine (0, 14, 28 days, *n* = 200), high dose vaccine (0, 14, 28 days, *n* = 199) or placebo (0, 21 days or 0, 14, 28 days, *n* = 160) (Fig. 1).

In phase 1 trial, all participants completed the full doses of vaccination and the planned visits within 28 days after the last dose vaccination. In phase 2 trial, 959 participants received the first dose vaccination, with 1 participant refused to receive vaccination after randomization, and 952 participants completed the full doses of vaccination and the planned visits within 30 days after the last dose vaccination. Baseline characteristics of the participants in both trials were comparable among groups (Table 1).

**Table 1:** Baseline characteristics

	Phase 1				Phase 2				
	Low dose (0, 28 days)	High dose (0, 28 days)	High dose (0, 14, 28 days)	Placebo	Low dose (0, 21 days)	High dose (0, 21 days)	Low dose (0, 14, 28 days)	High dose (0, 14, 28 days)	Placebo
*Adult group*	*n* = 24	*n* = 24	*n* = 24	*n* = 24	*n* = 100	*n* = 100	*n* = 100	*n* = 100	*n* = 80
Age, years	37.7 (8.7)	37.6 (8.1)	41.3 (7.2)	39.3 (7.1)	40.7 (11.1)	41.2 (11.8)	44.2 (9.9)	41.5 (11.1)	40.0 (10.3)
Sex									
Male	13 (54%)	11 (46%)	9 (38%)	15 (63%)	46 (46%)	41 (41%)	26 (26%)	42 (42%)	26 (26%)
Female	11 (46%)	13 (54%)	15 (63%)	9 (38%)	54 (54%)	59 (59%)	74 (74%)	58 (58%)	54 (54%)
Body-mass index, kg/m^2^	24.7 (3.1)	23.6 (2.6)	24.1 (2.9)	23.7 (2.7)	26.1 (4.0)	26.4 (4.3)	25.3 (3.7)	25.6 (3.8)	25.8 (4.3)
Underlying diseases									
Yes	2 (8%)	1 (4%)	6 (25%)	1 (4%)	13 (13%)	17 (17%)	6 (6%)	9 (9%)	9 (9%)
No	22 (92%)	23 (96%)	18 (75%)	23 (96%)	87 (87%)	83 (83%)	94 (94%)	91 (91%)	71 (71%)
*Elderly group*	*n* = 18	*n* = 18	*n* = 18	*n* = 18	*n* = 100	*n* = 100	*n* = 100	*n* = 99	*n* = 80
Age, years	63.3 (4.9)	64.1 (6.3)	63.4 (5.7)	64.3 (5.4)	66.7 (4.7)	66.7 (4.7)	67.7 (5.5)	67.1 (4.8)	67.0 (4.7)
Sex									
Male	9 (50%)	7 (39%)	6 (33%)	12 (67%)	53 (53%)	61 (61%)	62 (62%)	57 (57%)	46 (46%)
Female	9 (50%)	11 (61%)	12 (67%)	6 (33%)	47 (47%)	39 (39%)	38 (38%)	42 (42%)	34 (34%)
Body-mass index, kg/m^2^	24.9 (1.9)	24.8 (2.3)	24.6 (2.4)	24.0 (1.9)	26.5 (3.5)	26.1 (3.1)	25.6 (3.1)	25.7 (3.4)	26.2 (3.2)
Underlying diseases									
Yes	8 (44%)	8 (44%)	6 (33%)	8 (44%)	28 (28%)	34 (34%)	29 (29%)	31 (31%)	34 (34%)
No	10 (56%)	10 (56%)	12 (67%)	10 (56%)	72 (72%)	66 (66%)	71 (71%)	68 (68%)	46 (46%)

Data are number of participants (%) or mean (SD).

### Safety

In phase 1 trial, the most common solicited injection site adverse reaction within 7 days was pain (19 [20%] in the adult group, 6 [8%] in the elderly group), and the most common solicited systematic adverse reactions within 7 days were fatigue (5 [5%] in the adult group, 3 [4%] in the elderly group), cough (5 [5%] in the adult group, 2 [3%] in the elderly group) and sore throat (5 [5%] in the adult group, 2 [3%] in the elderly group) (Table 2). 48 (29%) of 168 participants reported at least one adverse reaction from the first dose vaccination to 28 days after the last dose vaccination, 7 (17%) in the low dose group (0, 28 days), 13 (31%) in the high dose group (0, 28 days), 15 (36%) in the high dose group (0, 14, 28 days), and 13 (31%) in the placebo group. Besides, compared with the placebo group, the incidences of abnormal changes in laboratory measurements in the vaccine groups were not significantly different (Supplementary Table 1). Unsolicited adverse reactions within 28 days after vaccination were comparable among different dose groups in both age groups (Supplementary Table 2).

**Table 2:** Adverse reactions within 7 days and overall adverse events within 28 days after vaccination in phase 1 trial

	Adult group					Elderly group				
	Low dose (0, 28 days *n* = 24)	High dose (0, 28 days *n* = 24)	High dose (0, 14, 28 days *n* = 24)	Placebo (*n* = 24)	*p* value	Low dose (0, 28 days *n* = 18)	High dose (0, 28 days *n* = 18)	High dose (0, 14, 28 days *n* = 18)	Placebo (*n* = 18)	*p* value
*Solicited adverse reactions within 7 days*										
Any	5 (21%)	7 (29%)	10 (42%)	7 (29%)	0.47	2 (11%)	3 (17%)	4 (22%)	5 (28%)	0.75
Grade ≥3	0 (0%)	0 (0%)	0 (0%)	0 (0%)	–	0 (0%)	0 (0%)	0 (0%)	0 (0%)	–
*Solicited injection site adverse reactions within 7 days*										
Any	3 (13%)	3 (13%)	8 (33%)	5 (21%)	0.23	1 (6%)	2 (11%)	3 (17%)	0 (0%)	0.50
Pain	3 (13%)	2 (8%)	8 (33%)	3 (13%)	0.12	1 (6%)	1 (6%)	3 (17%)	0 (0%)	0.38
Itch	0 (0%)	0 (0%)	0 (0%)	1 (4%)	>0.99	0 (0%)	1 (6%)	2 (11%)	0 (0%)	0.61
Redness	0 (0%)	0 (0%)	0 (0%)	1 (4%)	>0.99	–	–	–	–	–
Swelling	0 (0%)	1 (4%)	0 (0%)	0 (0%)	>0.99	–	–	–	–	–
*Solicited systematic adverse reactions within 7 days*										
Any	3 (13%)	5 (21%)	5 (21%)	4 (17%)	0.94	1 (6%)	2 (11%)	2 (11%)	5 (28%)	0.36
Fatigue	1 (4%)	2 (8%)	2 (8%)	0 (0%)	0.75	1 (6%)	1 (6%)	0 (0%)	1 (6%)	>0.99
Cough	1 (4%)	2 (8%)	0 (0%)	2 (8%)	0.75	0 (0%)	1 (6%)	0 (0%)	1 (6%)	>0.99
Sore throat	1 (4%)	1 (4%)	2 (8%)	1 (4%)	>0.99	0 (0%)	0 (0%)	2 (11%)	0 (0%)	0.24
Fever	1 (4%)	1 (4%)	2 (8%)	0 (0%)	0.90	0 (0%)	1 (6%)	0 (0%)	1 (6%)	>0.99
Headache	0 (0%)	0 (0%)	3 (13%)	1 (4%)	0.20	–	–	–	–	–
Nausea	0 (0%)	0 (0%)	2 (8%)	0 (0%)	0.24	0 (0%)	1 (6%)	0 (0%)	2 (11%)	0.24
Anorexia	0 (0%)	0 (0%)	1 (4%)	0 (0%)	>0.99	–	–	–	–	–
Joint pain	–	–	–	–	–	1 (6%)	0 (0%)	0 (0%)	0 (0%)	>0.99
*Adverse reactions within 7 days after the first dose*										
Any	2 (8%)	6 (25%)	7 (29%)	6 (25%)	0.31	1 (6%)	3 (17%)	2 (11%)	3 (17%)	0.86
Grade ≥3	0 (0%)	0 (0%)	0 (0%)	0 (0%)	–	0 (0%)	0 (0%)	0 (0%)	0 (0%)	–
*Adverse reactions within 7 days after the second dose*										
Any	4 (17%)	4 (17%)	5 (21%)	2 (8%)	0.76	1 (6%)	2 (11%)	2 (11%)	4 (22%)	0.60
Grade ≥3	0 (0%)	0 (0%)	0 (0%)	0 (0%)	–	0 (0%)	0 (0%)	0 (0%)	0 (0%)	–
*Adverse reactions within 7 days after the third dose*										
Any	–	–	5 (21%)	–	–	–	–	3 (17%)	–	–
Grade ≥3	–	–	0 (0%)	–	–	–	–	0 (0%)	–	–
*Overall adverse events within 28 days*										
Any	14 (58%)	13 (54%)	15 (63%)	15 (63%)	0.92	6 (33%)	5 (28%)	7 (39%)	8 (44%)	0.75
Grade ≥3	0 (0%)	0 (0%)	0 (0%)	0 (0%)	–	0 (0%)	0 (0%)	0 (0%)	0 (0%)	–

Data are *n* (%). Any refers to all the participants with any grade adverse reactions or events. Adverse reactions and events were graded according to the scale issued by the China State Food and Drug Administration.

In phase 2 trial, the most common solicited injection site adverse reaction within 7 days was also pain (53 [11%] in the adult group, 27 [6%] in the elderly group), and the most common solicited systematic adverse reactions within 7 days were cough (12 [3%] in the adult group, 7 [1%] in the elderly group), fever (10 [2%] in the adult group, 6 [1%] in the elderly group) and headache (9 [2%] in the adult group, 7 [1%] in the elderly group) (Table 3). 180 (19%) of 959 participants reported at least one adverse reaction from the first dose vaccination to 30 days after the last dose vaccination, 27 (14%) in the low dose group (0, 21 days), 38 (19%) in the high dose group (0, 21 days), 41 (21%) in the low dose group (0, 14, 28 days), 49 (25%) in the high dose group (0, 14, 28 days), and 24 (15%) in the placebo group. Unsolicited adverse reactions within 30 days after vaccination were comparable among different dose groups in both age groups (Supplementary Table 3).

**Table 3:** Adverse reactions within 7 days and overall adverse events within 30 days after vaccination in phase 2 trial

	Adult group						Elderly group					
	Low dose (0, 21 days *n* = 100)	High dose (0, 21 days *n* = 100)	Low dose (0, 14, 28 days *n* = 100)	High dose (0, 14, 28 days *n* = 100)	Placebo (*n* = 80)	*p* value	Low dose (0, 21 days *n* = 100)	High dose (0, 21 days *n* = 100)	Low dose (0, 14, 28 days *n* = 100)	High dose (0, 14, 28 days *n* = 99)	Placebo (*n* = 80)	*p* value
*Solicited adverse reactions within 7 days*												
Any	16 (16%)	19 (19%)	22 (22%)	30 (30%)	16 (20%)	0.16	10 (10%)	12 (12%)	18 (18%)	16 (16%)	3 (4%)	0.04
Grade ≥3	0 (0%)	0 (0%)	0 (0%)	1 (1%)	0 (0%)	>0.99	0 (0%)	0 (0%)	0 (0%)	0 (0%)	0 (0%)	–
*Solicited injection site adverse reactions within 7 days*												
Any	6 (6%)	16 (16%)	13 (13%)	25 (25%)	11 (14%)	0.01	4 (4%)	9 (9%)	10 (10%)	8 (8%)	1 (1%)	0.10
Pain	6 (6%)	15 (15%)	6 (6%)	16 (16%)	10 (13%)	0.05	4 (4%)	9 (9%)	8 (8%)	5 (5%)	1 (1%)	0.16
Itch	0 (0%)	1 (1%)	8 (8%)	13 (13%)	1 (1%)	<0.01	0 (0%)	1 (1%)	3 (3%)	3 (3%)	0 (0%)	0.20
Swelling	0 (0%)	0 (0%)	7 (7%)	4 (4%)	0 (0%)	<0.01	–	–	–	–	–	–
Induration	0 (0%)	1 (1%)	3 (3%)	3 (3%)	1 (1%)	0.40	–	–	–	–	–	–
Redness	0 (0%)	0 (0%)	3 (3%)	3 (3%)	0 (0%)	0.05	–	–	–	–	–	–
Rash	0 (0%)	0 (0%)	1 (1%)	0 (0%)	0 (0%)	>0.99	–	–	–	–	–	–
*Solicited systematic adverse reactions within 7 days*												
Any	12 (12%)	7 (7%)	10 (10%)	9 (9%)	6 (8%)	0.76	6 (6%)	3 (3%)	12 (12%)	10 (10%)	2 (3%)	0.03
Cough	3 (3%)	0 (0%)	2 (2%)	3 (3%)	4 (5%)	0.25	1 (1%)	0 (0%)	3 (3%)	2 (2%)	1 (1%)	0.47
Fatigue	2 (2%)	2 (2%)	5 (5%)	1 (1%)	0 (0%)	0.22	1 (1%)	0 (0%)	1 (1%)	1 (1%)	0 (0%)	0.95
Fever	4 (4%)	2 (2%)	2 (2%)	2 (2%)	0 (0%)	0.52	0 (0%)	1 (1%)	2 (2%)	3 (3%)	0 (0%)	0.27
Headache	3 (3%)	2 (2%)	3 (3%)	1 (1%)	0 (0%)	0.58	1 (1%)	1 (1%)	2 (2%)	3 (3%)	0 (0%)	0.64
Sore throat	0 (0%)	0 (0%)	3 (3%)	3 (3%)	2 (3%)	0.14	2 (2%)	1 (1%)	1 (1%)	0 (0%)	0 (0%)	0.81
Nausea	2 (2%)	4 (4%)	1 (1%)	1 (1%)	0 (0%)	0.33	0 (0%)	0 (0%)	3 (3%)	2 (2%)	1 (1%)	0.19
Joint pain	0 (0%)	0 (0%)	2 (2%)	2 (2%)	1 (1%)	0.44	0 (0%)	0 (0%)	1 (1%)	2 (2%)	0 (0%)	0.28
Diarrhea	3 (3%)	0 (0%)	1 (1%)	0 (0%)	0 (0%)	0.16	1 (1%)	0 (0%)	0 (0%)	0 (0%)	0 (0%)	>0.99
Vomit	–	–	–	–	–	–	0 (0%)	0 (0%)	2 (2%)	1 (1%)	0 (0%)	0.36
Anorexia	–	–	–	–	–	–	1 (1%)	0 (0%)	1 (1%)	0 (0%)	0 (0%)	>0.99
*Adverse reactions within 7 days after the first dose*												
Any	13 (13%)	20 (20%)	4 (4%)	11 (11%)	9 (11%)	0.01	8 (8%)	8 (8%)	7 (7%)	7 (7%)	4 (5%)	0.94
Grade ≥3	0 (0%)	0 (0%)	0 (0%)	0 (0%)	0 (0%)	–	0 (0%)	0 (0%)	0 (0%)	0 (0%)	0 (0%)	–
*Adverse reactions within 7 days after the second dose*												
Any	5 (5%)	9 (9%)	5 (5%)	11 (11%)	7 (9%)	0.41	4 (4%)	6 (6%)	7 (7%)	6 (6%)	3 (4%)	0.83
Grade ≥3	0 (0%)	0 (0%)	0 (0%)	0 (0%)	0 (0%)	–	0 (0%)	0 (0%)	0 (0%)	0 (0%)	0 (0%)	–
*Adverse reactions within 7 days after the third dose*												
Any	–	–	16 (16%)	21 (21%)	–	–	–	–	8 (8%)	9 (9%)	–	–
Grade ≥3	–	–	0 (0%)	0 (0%)	–	–	–	–	0 (0%)	0 (0%)	–	–
*Overall adverse events within 30 days*												
Any	30 (30%)	37 (37%)	31 (31%)	41 (41%)	23 (29%)	0.32	19 (19%)	26 (26%)	32 (32%)	28 (28%)	14 (18%)	0.11
Grade ≥3	0 (0%)	0 (0%)	0 (0%)	0 (0%)	0 (0%)	–	0 (0%)	0 (0%)	0 (0%)	0 (0%)	0 (0%)	–

Data are *n* (%). Any refers to all the participants with any grade adverse reactions or events. Adverse reactions and events were graded according to the scale issued by the China State Food and Drug Administration.

In phase 1 trial, no significant difference was found in the overall adverse reactions among groups, while the overall number of adverse reactions in phase 2 trial was higher in the high dose group (0, 14, 28 days) than that in the placebo group. Most adverse reactions were mild or moderate in severity. In both trials, no significant difference was observed in the incidences of overall adverse events among groups. No vaccine related serious adverse events were documented from the first dose vaccination to 28 days after the last dose vaccination in phase 1 trial. In phase 2 trial, most adverse reactions were mild or moderate in severity, with only 1 participant had grade 3 injection site adverse reactions (redness and swelling) and recovered with no treatment within 3 days (supplementary table 4).

### Humoral immunogenicity

In phase 1 trial, all three vaccine groups showed rapid binding antibody responses to RBD in from 14 days after the last dose vaccination (Table 4). At 28 days after the last dose vaccination, binding antibody of the adult participants in the high dose group (0, 14, 28 days) was higher, with the geometric mean titre of 1282.1 (95% CI 713.6–2303.4), followed by 96.9 (55.4–169.4) in the high dose group (0, 28 days), 39.3 (25.2–61.2) in the low dose group (0, 28 days), and 20.0 (20.0–20.0) in the placebo group. At 28 days after the last dose vaccination, binding antibody of the older participants in the high dose group (0, 14, 28 days) was higher, with the geometric mean titre of 548.2 (95% CI 198.2–1516.6), followed by 45.1 (26.8–76.0) in the high dose group (0, 28 days), 42.9 (20.1–91.6) in the low dose group (0, 28 days), and 21.7 (18.2–25.9) in the placebo group. Seroconversion of anti-RBD antibodies was observed in 23 (96%) of 24 adult participants in the high dose group (0, 14, 28 days), and 14 (78%) of 18 older participants in the high dose group (0, 14, 28 days).

**Table 4:** Specific antibody responses to RBD, neutralising antibodies to live SARS-CV-2 and pseudovirus post vaccination in phase 1 trial

	Adult group					Elderly group				
	Low dose (0, 28 days *n* = 24)	High dose (0, 28 days *n* = 24)	High dose (0, 14, 28 days *n* = 24)	Placebo (*n* = 24)	*p* value	Low dose (0, 28 days *n* = 18)	High dose (0, 28 days *n* = 18)	High dose (0, 14, 28 days *n* = 18)	Placebo (*n* = 18)	*p* value
Day 0										
*ELISA antibodies to the receptor binding domain*										
GMT	20.0 (20.0–20.0)	20.9 (19.1–22.8)	20.8 (19.2–22.5)	20.0 (20.0–20.0)	0.57	20.0 (20.0–20.0)	20.0 (20.0–20.0)	20.0 (20.0–20.0)	20.0 (20.0–20.0)	–
*Neutralising antibodies to live SARS-CoV-2*										
GMT	0.7 (0.7–0.7)	0.7 (0.7–0.7)	0.7 (0.7–0.7)	0.7 (0.7–0.7)	–	0.7 (0.7–0.7)	0.7 (0.7–0.7)	0.7 (0.7–0.7)	0.7 (0.7–0.7)	–
*Neutralising antibodies to pseudovirus*										
GMT	17.2 (14.7–20.1)	16.0 (14.0–18.2)	16.1 (14.5–17.9)	15.5 (14.5–16.6)	0.64	15.7 (14.3–17.2)	15.0 (15.0–15.0)	15.0 (15.0–15.0)	15.0 (15.0–15.0)	0.40
Day 7										
*ELISA antibodies to the receptor binding domain*										
GMT	21.0 (19.0–23.1)	23.2 (19.6–27.5)	402.6 (221.3–732.7)	20.0 (20.0–20.0)	<0.001	24.0 (16.3–35.3)	21.3 (18.7–24.2)	101.2 (40.8–250.7)	20.0 (20.0–20.0)	<0.001
Seroconversion	0 (0%)	0 (0%)	22 (92%)	0 (0%)	<0.001	1 (6%)	0 (0%)	9 (50%)	0 (0%)	<0.001
*Neutralising antibodies to live SARS-CoV-2*										
GMT	0.8 (0.7–1.0)	0.9 (0.8–1.2)	19.4 (11.7–32.2)	0.7 (0.7–0.7)	<0.001	0.8 (0.7–1.0)	1.0 (0.8–1.3)	4.5 (2.2–9.0)	0.7 (0.7–0.7)	<0.001
Seroconversion	0 (0%)	0 (0%)	23 (96%)	0 (0%)	<0.001	0 (0%)	0 (0%)	11 (61%)	0 (0%)	<0.001
*Neutralising antibodies to pseudovirus*										
GMT	17.6 (14.6–21.2)	19.7 (15.9–24.5)	82.1 (56.0–120.5)	15.0 (15.0–15.0)	<0.001	16.9 (14.2–20.1)	16.1 (13.9–18.5)	21.9 (16.4–29.4)	15.0 (15.0–15.0)	0.018
Seroconversion	3 (13%)	6 (25%)	21 (88%)	0 (0%)	<0.001	2 (11%)	1 (6%)	6 (33%)	0 (0%)	0.023
Day 14										
*ELISA antibodies to the receptor binding domain*										
GMT	31.3 (21.9–44.7)	62.7 (37.5–104.8)	1348.8 (654.5–2779.5)	20.0 (20.0–20.0)	<0.001	34.1 (19.2–60.8)	25.3 (19.3–33.2)	268.7 (113.9–634.2)	20.0 (20.0–20.0)	<0.001
Seroconversion	5 (21%)	10 (42%)	22 (92%)	0 (0%)	<0.001	3 (17%)	2 (11%)	14 (78%)	0 (0%)	<0.001
*Neutralising antibodies to live SARS-CoV-2*										
GMT	1.0 (0.8–1.3)	1.5 (1.0–2.2)	28.2 (17.5–45.4)	0.7 (0.7–0.7)	<0.001	0.9 (0.7–1.2)	1.0 (0.8–1.4)	7.1 (3.2–15.7)	0.7 (0.7–0.7)	<0.001
Seroconversion	1 (4%)	3 (13%)	23 (96%)	0 (0%)	<0.001	0 (0%)	0 (0%)	12 (67%)	0 (0%)	<0.001
*Neutralising antibodies to pseudovirus*										
GMT	17.0 (14.7–19.6)	18.6 (15.0–23.0)	86.7 (58.7–128.0)	15.5 (14.5–16.5)	<0.001	15.7 (14.2–17.4)	21.9 (16.6–29.1)	25.6 (19.6–33.5)	15.7 (14.2–17.4)	<0.001
Seroconversion	3 (13%)	4 (17%)	23 (96%)	1 (4%)	<0.001	1 (6%)	6 (33%)	10 (56%)	1 (6%)	<0.001
Day 28										
*ELISA antibodies to the receptor binding domain*										
GMT	39.3 (25.2–61.2)	96.9 (55.4–169.4)	1282.1 (713.6–2303.4)	20.0 (20.0–20.0)	<0.001	42.9 (20.1–91.6)	45.1 (26.8–76.0)	548.2 (198.2–1516.6)	21.7 (18.2–25.9)	<0.001
Seroconversion	7 (29%)	14 (58%)	23 (96%)	0 (0%)	<0.001	5 (28%)	5 (28%)	14 (78%)	1 (6%)	<0.001
*Neutralising antibodies to live SARS-CoV-2*										
GMT	1.2 (0.9–1.6)	2.3 (1.5–3.3)	102.9 (61.9–171.2)	0.7 (0.7–0.7)	<0.001	1.0 (0.8–1.2)	1.5 (1.1–2.0)	22.4 (7.7–65.5)	0.7 (0.7–0.7)	<0.001
Seroconversion	1 (4%)	7 (29%)	24 (100%)	0 (0%)	<0.001	0 (0%)	1 (6%)	14 (78%)	0 (0%)	<0.001
*Neutralising antibodies to pseudovirus*										
GMT	26.5 (19.8–35.5)	29.0 (21.5–39.1)	108.0 (78.8–148.2)	15.5 (14.4–16.7)	<0.001	19.4 (15.7–24.0)	25.7 (19.4–34.2)	50.5 (36.3–70.1)	16.5 (13.5–20.1)	<0.001
Seroconversion	10 (42%)	12 (50%)	23 (96%)	1 (4%)	<0.001	5 (28%)	9 (50%)	15 (83%)	1 (6%)	<0.001

In phase 2 trial, rapid binding antibody responses to RBD were also observed from 14 days after the last dose vaccination, and peaked at 30 days after the last dose vaccination (Table 5). At 30 days after the last dose vaccination, binding antibody of the adult participants in the high dose group (0, 14, 28 days) was higher, with the geometric mean titre of 1099.9 (95% CI 812.6–1488.7), followed by 156.2 (112.1–217.6) in the low dose group (0, 14, 28 days), 135.9 (91.9–200.8) in the high dose group (0, 21 days), 40.0 (30.6–52.3) in the low dose group (0, 21 days), and 21.8 (19.6–24.2) in the placebo group. At 30 days after the last dose vaccination, binding antibody of the older participants in the high dose group (0, 14, 28 days) was higher, with the geometric mean titre of 333.2 (95% CI 232.6–477.3), followed by 54.3 (40.3–73.3) in the low dose group (0, 14, 28 days), 54.4 (39.7–74.7) in the high dose group (0, 21 days), 22.5 (20.2–25.1) in the high dose group (0, 21 days), and 20.8 (19.7–22.1) in the placebo group. Seroconversion of anti-RBD antibodies was noted in 97 (99%) of 99 adult participants in the high dose group (0, 14, 28 days), 78 (80%) of 98 older participants in the high dose group (0, 14, 28 days).

**Table 5:** Specific antibody responses to RBD and neutralising antibodies to live SARS-CV-2 post vaccination in phase 2 trial

	Adult group						Elderly group					
	Low dose (0, 21 days *n* = 99)	High dose (0, 21 days *n* = 100)	low dose (0, 14, 28 days *n* = 100)	High dose (0, 14, 28 days *n* = 99)	Placebo (*n* = 79)	*p* value	Low dose (0, 21 days *n* = 99)	High dose (0, 21 days *n* = 99)	low dose (0, 14, 28 days *n* = 99)	High dose (0, 14, 28 days *n* = 98)	Placebo (*n* = 80)	*p* value
Day 0												
*ELISA antibodies to the receptor binding domain*												
GMT	20.0 (20.0–20.0)	21.1 (19.5–22.9)	20.0 (20.0–20.0)	20.4 (19.6–21.1)	20.7 (19.3–22.2)	0.44	20.0 (20.0–20.0)	21.1 (19.6–22.7)	21.0 (19.4–22.8)	21.8 (19.7–24.1)	20.4 (19.6–21.2)	0.49
*Neutralising antibodies to live SARS-CoV-2*												
GMT	0.7 (0.7–0.7)	0.7 (0.7–0.7)	0.7 (0.7–0.7)	0.7 (0.7–0.7)	0.7 (0.7–0.7)	–	0.7 (0.7–0.7)	0.7 (0.7–0.7)	0.7 (0.7–0.7)	0.7 (0.7–0.7)	0.7 (0.7–0.7)	–
Day 14												
*ELISA antibodies to the receptor binding domain*												
GMT	32.1 (25.2–41.0)	93.2 (63.6–136.4)	105.7 (76.4–146.3)	601.2 (441.5–818.8)	20.8 (19.3–22.4)	<0.001	21.2 (19.8–22.7)	38.1 (29.2–49.8)	44.1 (34.1–57.1)	198.3 (140.4–280.1)	21.2 (19.5–23.0)	<0.001
Seroconversion	15 (25%)	49 (49%)	55 (55%)	89 (89%)	0 (0%)	<0.001	3 (3%)	20 (20%)	26 (26%)	68 (69%)	1 (1%)	<0.001
*Neutralising antibodies to live SARS-CoV-2*												
GMT	1.0 (0.9–1.2)	2.2 (1.7–2.9)	3.4 (2.5–4.7)	25.7 (19.3–34.3)	0.7 (0.7–0.7)	<0.001	1.0 (0.9–1.1)	1.1 (0.9–1.1)	1.2 (1.0–1.4)	7.3 (5.2–10.2)	0.7 (0.7–0.7)	<0.001
Seroconversion	7 (7%)	18 (18%)	49 (49%)	91 (92%)	0 (0%)	<0.001	1 (1%)	4 (4%)	9 (9%)	66 (67%)	0 (0%)	<0.001
Day 30												
*ELISA antibodies to the receptor binding domain*												
GMT	40.0 (30.6–52.3)	135.9 (91.9–200.8)	156.2 (112.1–217.6)	1099.9 (812.6–1488.7)	21.8 (19.6–24.2)	<0.001	22.5 (20.2–25.1)	54.4 (39.7–74.7)	54.3 (40.3–73.3)	333.2 (232.6–477.3)	20.8 (19.7–22.1)	<0.001
Seroconversion	26 (26%)	57 (58%)	69 (69%)	97 (99%)	1 (1%)	<0.001	5 (5%)	31 (31%)	33 (34%)	78 (80%)	1 (1%)	<0.001
*Neutralising antibodies to live SARS-CoV-2*												
GMT	1.9 (1.6–2.4)	3.6 (2.6–4.9)	8.1 (5.9–11.2)	102.6 (75.2–140.1)	0.7 (0.7–0.7)	<0.001	1.3 (1.2–1.5)	1.6 (1.4–1.9)	1.7 (1.4–2.1)	21.3 (13.8–33.1)	0.7 (0.7–0.7)	<0.001
Seroconversion	17 (17%)	37 (37%)	73 (73%)	94 (96%)	0 (0%)	<0.001	4 (4%)	11 (11%)	18 (18%)	72 (73%)	0 (0%)	<0.001

In phase 1 trial, neutralising antibody responses to live SARS-CoV-2 (50TCID_50_) were observed in the high dose group (0, 14, 28 days) from 7 days after the last dose vaccination, peaking at 28 days after the last dose vaccination, while those in the low dose group (0, 28 days) and the high dose group (0, 28 days) were still weak by 28 days after the last dose vaccination (Table 4). In the adult group, the geometric mean titre of neutralising antibody was 102.9 (95% CI 61.9–171.2) in the high dose group (0, 14, 28 days), which was significantly higher compared with 2.2 (1.5–3.3) in the high dose group (0, 28 days), 1.2 (0.9–21.6) in the low dose group (0, 28 days), and 0.7 (0.7–0.7) in the placebo group. In the elderly group, the geometric mean titre of neutralising antibody was 22.4 (95% CI 7.7–65.5) in the high dose group (0, 14, 28 days), which was significantly higher compared with 1.5 (1.1–2.0) in the high dose group (0, 28 days), 1.0 (0.8–1.2) in the low dose group (0, 28 days), and 0.7 (0.7–0.7) in the placebo group. Meanwhile, 24 (100%) of 24 adult participants and 14 (78%) of 18 older participants in the high dose group (0, 14, 28 days) had seroconversion in neutralising antibody titres against live SARS-CoV-2.

In phase 2 trial, neutralising antibody responses to live SARS-CoV-2 (50TCID_50_) were observed in the high dose group (0, 14, 28 days) from 14 days after the last dose vaccination, peaking at 28 days after the last dose vaccination, while those in the low dose group (0, 21 days), the high dose group (0, 21 days) and the low dose group (0, 14, 28 days) were still weak by 28 days after the last dose vaccination (Table 5). In the adult group, the geometric mean titre of neutralising antibody was 102.6 (95% CI 75.2–140.1) in the high dose group (0, 14, 28 days), which was significantly higher compared with 8.1 (5.9–11.2) in the low dose group (0, 14, 28 days), 3.6 (2.6–4.9) in the high dose group (0, 21 days), 1.9 (1.6–2.4) in the low dose group (0, 21 days), and 0.7 (0.7–0.7) in the placebo group. In the elderly group, the geometric mean titre of neutralising antibody was 21.3 (95% CI 13.8–33.1) in the high dose group (0, 14, 28 days), which was significantly higher compared with 1.7 (1.4–2.1) in the low dose group (0, 14, 28 days), 1.6 (1.4–1.9) in the high dose group (0, 21 days), 1.3 (1.2–1.5) in the low dose group (0, 21 days), and 0.7 (0.7–0.7) in the placebo group. Meanwhile, 94 (96%) of 99 adult participants and 72 (73%) of 98 older participants in the high dose group (0, 14, 28 days) had seroconversion in neutralising antibody titres against live SARS-CoV-2.

In phase 1 trial, similar patterns of the neutralising antibody titres against pseudovirus post-vaccination among groups were also noted (Table 4). In the adult group, the geometric mean titre of neutralising antibody was 108.0 (95% CI 78.8–148.2) in the high dose group (0, 14, 28 days), and 23 (96%) of 24 participants in the high dose group (0, 14, 28 days) had seroconversion at 28 days after the last dose vaccination. In the elderly group, the geometric mean titre of neutralising antibody was 50.5 (95% CI 36.3–70.1) in the high dose group (0, 14, 28 days), and 15 (83%) of 18 participants in the high dose group (0, 14, 28 days) had seroconversion at 28 days after the last dose vaccination.

### Cellular immunogenicity

**Fig. 2:**
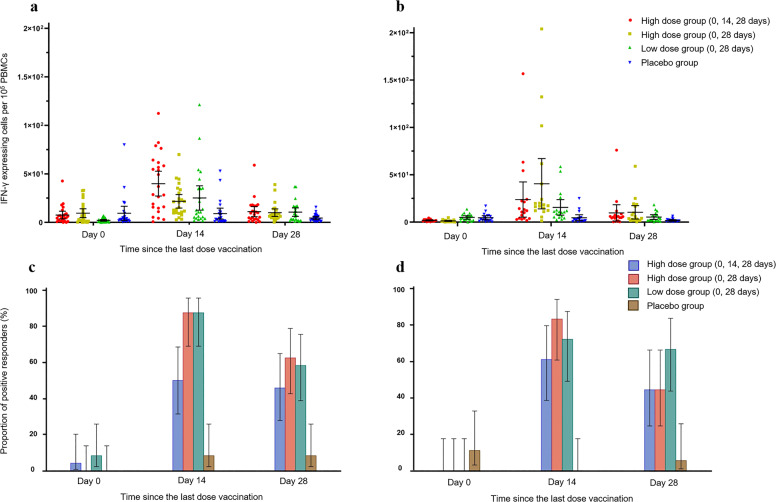
Specific T-cell response measured by ELISpot in phase 1 trial. In phase 1 trial, level of IFN-γ was detected by ELISpot before the first dose vaccination, 14 and 28 days after the last dose vaccination (**a**, **b**), and then the positive rate of IFN-γ was calculated (**c**, **d**). Data are presented as mean and 95% CI. **a**, **c**: Adult group. **b**, **d**: Elderly group

In phase 1 trial, the level of IFN-γ measured by ELISpot was higher at 14 days than that at 28 days after the last dose vaccination (Fig. 2), with a mean number of spot-forming cells per 100,000 cells of 25.2 (95% CI 12.6–37.7) in the adult low dose group (0, 28 days), 21.8 (14.8–28.7) in the adult high dose group (0, 28 days), 39.9 (27.2–52.7) in the adult high dose group (0, 14, 28 days), and 9.1 (3.5–14.7) in the adult placebo group. The corresponding results in the elderly group were 15.5 (95% CI 7.3–23.7), 40.5 (13.9–67.1), 23.8 (5.3–42.4) and 4.6 (1.5–7.7), respectively. The positive rate of IFN-γ measured by ELISpot also peaked at 14 days after the last dose vaccination, and that among three vaccine groups were not significantly different, with 50% in the adult low dose group (0, 28 days), 88% in the adult high dose group (0, 28 days), 88% in the adult high dose group (0, 14, 28 days), and 8% in the adult placebo group. The corresponding results in the elderly group were 61%, 83%, 72% and 0%, respectively.

## Discussion

As far as we know, our study is the first report phase 1 and phase 2 clinical trials for an RBD-based protein subunit COVID-19 vaccine including the elderly participants, and also achieved acceptable immunogenicity in them. In this study, we evaluated the safety and immunogenicity of the recombinant COVID-19 vaccine (Sf9 cells) with different dosages and vaccination schedules. This vaccine was made by adding an adjuvant of aluminium hydroxide, which was a tradition adjuvant. This adjuvant had limited ability to improve the immunogenicity of the vaccine, but it was relatively safe compared to novel adjuvants, such as MF59, which is more locally reactogenic. Take the influenza vaccine as an example, addition of MF59 increased the immunogenicity in the elderly to a certain extent, but it also increased the incidence of adverse reactions accordingly.^9,10^ In the case of using the aluminium adjuvant, the immunogenicity of our vaccine is still relatively good, we hypothesized that might be attributed to the expression of insect cells.

Baculovirus expression vector system (BEVS) has been used in the expression of a variety of proteins, and provides convenient and sufficient materials for vaccine research and development. BEVS has several merits. First, baculoviruses only parasitize invertebrates, and their products are highly safe to mammals. Second, the recombinant protein will express at high level in BEVS. Third, baculovirus can correctly fold and translate the recombinant protein and then modify it to obtain the protein with biological activity. Up to now, BEVS has been applied in two approved vaccines for human use, the influenza vaccine manufactured by Protein Sciences Corporation^11^ and the Human papillomavirus vaccine manufactured by GSK.^12^

We report the first analysis results of the phase 1 and phase 2 trials of a recombinant COVID-19 vaccine (Sf9 cells), the results showed this vaccine was safe and immunogenic in healthy population aged 18 years or older in China. The phase 2 trial had been conducted before we get the immunogenicity results of the phase 1 trial, and the vaccine dosage in the phase 2 trial was chose mainly according to the safety data. In terms of safety, the recombinant COVID-19 vaccine (Sf9 cells) was safe and well-tolerated in all age and dose groups. The incidence of adverse reactions within 7 days after each dose vaccination of our vaccine was no more than 21%, which was much lower than that of another protein subunit COVID-19 vaccine developed by Novavax.^13^ The most common solicited injection site adverse reaction in both trials was pain, and the most common solicited systematic adverse reactions were fatigue, cough and sore throat in phase 1 trial, and cough, fever and headache in phase 2 trial. The incidence rates of adverse reactions to the recombinant COVID-19 vaccine (Sf9 cells) were generally comparable across different vaccine groups with no clear dose–response associations and were similar to the placebo group, indicating that the adverse reactions may not be entirely due to the vaccine but may be because of intramuscular injections or the aluminium hydroxide adjuvant that contained in both the vaccine and placebo. Besides, the adverse reactions within 7 days and overall adverse events within 28 or 30 days after vaccination in the elderly group were much lower than those in the adult group in both trials, which showed the safety of the recombinant COVID-19 vaccine (Sf9 cells) in the elderly was more acceptable than the adults. Moreover, the adverse reactions within 7 days and overall adverse events within 30 days after vaccination in phase 2 trial was lower than those in phase 1 trial, these may be more stable results caused by the larger sample size. Additionally, laboratory measurements showed no abnormal changes associated with the vaccine.

The results of rapid binding antibody and neutralising antibody revealed that high dose (40 μg) of the recombinant COVID-19 vaccine (Sf9 cells) with the schedule of 0, 14, 28 days had good immunogenicity, especially in adult participants, and its immunogenicity in older participants was also acceptable. A detectable immune response begins quickly, with antibodies peaking at 28 days or 30 days after the last dose vaccination and T cell responses peaking at 14 days after the last dose vaccination. Three doses of high dose recombinant COVID-19 vaccine (Sf9 cells) could induce a four-fold increase in binding antibodies to RBD in 96%-99% of adult participants, and a four-fold increase in neutralising antibodies to live virus in 96–100% of adult participants. Even in older participants, the four-fold increase rates in binding antibodies to RBD and neutralising antibodies to live virus were 78%–80% and 73%–78%, respectively. Although the seroconversion rate of rapid binding antibody and neutralising antibody in high dose group (0, 28 days) were significantly lower than those in high dose vaccine (0, 14, 28 days), no significant difference was found in the positive rate of IFN-γ at 14 days after the last dose vaccination between the two groups in phase 1 trial. Compared with another protein subunit COVID-19 vaccine manufactured by Anhui Zhifei Longcom Biopharmaceutical,^14^ which has been permitted for emergency use in China, the immunogenicity of 40 μg recombinant COVID-19 vaccine (Sf9 cells) with the schedule of 0, 14, 28 days was comparable with that of their vaccine. However, their vaccine takes at least two months to complete the full schedule, which is two folds higher than ours. Thus, the schedule of our vaccine will be superior for emergency use.

The phase 3 trial results of the COVID-19 vaccine developed by Novavax reflected promising prospect of the protein subunit COVID-19 vaccine. Other protein subunit COVID-19 vaccines developed by various manufacturers are demanded to cope with the problem of insufficient supply. Among the clinical trials of three protein subunit vaccines, only one protein subunit vaccine enrolled both adults and the elderly as the participants (SCB-2019), the other two protein subunit vaccines (NVX-CoV2373 and ZF2001) only enrolled adult participants. The enrollment of the elderly is one of the highlights of our study, al. Our findings in phase 1 and phase 2 trials showed that the recombinant COVID-19 vaccine (Sf9 cells) may be a good candidate vaccine. Currently, the phase 1 and 2 trials are still ongoing, and we will continue to observe the long-term immunogenicity of the recombinant COVID-19 vaccine (Sf9 cells) for 1 year.

Our trials have some strengths. For example, we included relatively large numbers of elderly participants, which overcame the problem of too few older individuals in other COVID-19 vaccine trials. Besides, no participant was exposed to SARS-CoV-2 subsequently, which indicated that all immunogenicity results were from the vaccine, not extraneous infection. There are also several limitations of our studies. First, no children were enrolled in our trials. Second, we did not detect cellular immunogenicity in phase 2 trial due to the large sample size and the workload, and the cellular immunogenicity index in phase 1 trial was relatively simple, only IFN-γ was detected. Third, similar to other COVID-19 vaccine,^15,16^ we found that older participants had significantly lower immune responses, but higher safety to the recombinant COVID-19 vaccine (Sf9 cells) than adult participants, and the neutralizing antibody of older participants in our study was higher than that in the adenovirus vectored vaccine. Therefore, an additional dose or higher vaccine dosage may be essential for the older population to induce a better immune response, which requires further study.

In conclusion, we found that the recombinant COVID-19 vaccine (Sf9 cells) is safe and immunogenic in healthy population aged 18 years or older. Specific humoral responses against SARS-CoV-2 peaked at nearly 1 month after three doses vaccination. The results support testing of the recombinant COVID-19 vaccine (Sf9 cells) at high dose with three doses schedule in a phase 3 efficacy trial. We will continue the observation of one-year immune persistence in the phase 1 and 2 trials, and conduct a global phase III clinical trial to further evaluate the efficacy of the recombinant COVID-19 vaccine (Sf9 cells) in adults aged 18 years and older.

## Materials and methods

### Study design and participants

We did two single-center, randomised, double-blind, placebo-controlled phase 1 and phase 2 trials of a recombinant COVID-19 vaccine (Sf9 cells) in Taizhou and She yang, Jiangsu province, China, respectively. These two studies were undertaken by Jiangsu Provincial Center for Disease Control and Prevention, and were conducted according to the Good Clinical Practice and Declaration of Helsinki. The protocols were approved by the National Medical Products Administration, China, and were reviewed by the institutional review board of the Jiangsu Provincial Center for Disease Control and Prevention.

Eligible participants in both trials were healthy population aged 18 years or older, who signed the informed consent, had negative HIV and SARS-CoV-2 (IgG and IgM) antibody testing results confirmed by rapid test kit using fingertip blood. Besides, a negative throat swab for SARS-CoV-2 nucleic acid, a clear chest CT image free from imaging features of COVID-19, and no significantly abnormal blood routine, blood biochemical, coagulation function and urine routine test results were also needed for inclusion in phase 1 trial. Pregnant and breastfeeding women, people with allergic history, convulsion, epilepsy, encephalopathy, mental illness, acute infection, severe and uncontrollable cardiovascular disease or other chronic illnesses were excluded. Complete lists of the inclusion and exclusion criteria could be found in the protocols.

### Randomization and masking

The recombinant COVID-19 vaccine (Sf9 cells) was developed by West China Hospital of Sichuan University. The vaccine is a baculovirus vectored vaccine expressing SARS-CoV-2 spike protein receptor binding domain (S-RBD) in Sf9 cells, and adjuvanted with aluminium hydroxide. The placebos are consistent with the experimental vaccines except for the vaccine antigen, and they are also identical in appearance. The stratified block randomization and intragroup double-blind design are adopted in both studies. The candidate vaccine and placebo control within each dose group are set at a ratio of 3:1 (phase 1) or 5:1 (phase 2). Group allocation was not known to investigators, participants and biological sample testing personnel.

### Procedures

Two age groups (adult and elderly groups) were enrolled in both studies. In phase 1 trial, participants in each age group were sequentially enrolled in a dose-escalating manner with two immunisation schedules, including low dose group (0, 28 days), high dose group (0, 28 days), and high dose group (0, 14, 28 days). In the adult group in phase 1 trial, the first 32 participants were allocated to receive the low dose vaccine (20 μg), or placebo at a ratio of 3:1 with the schedule of 0, 28 days, after safety observation for 7 days, participants were allocated to receive the high dose vaccine (40 μg) or placebo at a ratio of 3:1 with the schedule of 0, 28 days and 0, 14, 28 days simultaneously, each immunization schedule involved 32 participants. The elderly group in phase 1 trial was enrolled when the safety is confirmed 7 days after the first dose in the adult high dose group. In elderly group in phase 1 trial, the first 24 participants were allocated to receive the low dose vaccine (20 μg) or placebo at a ratio of 3:1 with the schedule of 0, 28 days, after safety observation for 7 days, participants were allocated to receive the high dose vaccine (40 μg) or placebo at a ratio of 3:1 with the schedule of 0, 28 days and 0, 14, 28 days simultaneously, each immunization schedule involved 24 participants. During the dose-escalation process, the administration of higher dosage vaccine was paused if any of the study suspension criteria was met. In phase 2 trial, participants were enrolled simultaneously with two immunisation schedules, including low dose group (0, 21 days), high dose group (0, 21 days), low dose group (0, 14, 28 days) and high dose group (0, 14, 28 days), the candidate vaccine and placebo control within each dose group are set at a ratio of 5:1. The adverse events and abnormal changes in laboratory measurements were graded in line with the standard released by the National Medical Products Administration, China. (version 2019).^17^

### Outcomes

The study objectives were to evaluate the safety and immunogenicity of recombinant COVID-19 vaccine (Sf9 cells). The primary endpoint in phase 1 trial was the incidence of adverse reactions within 7 days after each dose vaccination. The secondary endpoints in phase 1 trial for safety were the incidence of adverse events and serious adverse events from the first dose to 28 days after the last dose, and abnormal changes in laboratory measures at 3 days after each dose vaccination. The secondary endpoints for humoral immunogenicity in phase 1 trial were the geometric mean titre (GMT), seroconversion rate (the titre after vaccination increased by at least 4 times from baseline), and geometric mean fold increase (GMI) of RBD-specific ELISA antibody responses at 7 days, 14 days and 28 days after the last dose vaccination, and those of neutralising antibody responses against live virus or pseudovirus. Besides, the secondary endpoints for cellular immunogenicity in phase 1 trial were positive rate and level of interferon γ (IFN-γ) 14 and 28 days after the last dose vaccination, measured by enzyme-linked immunospot (ELISpot).

The primary endpoints in phase 2 trial were the GMT of RBD-specific ELISA antibody responses at 30 days after the last dose vaccination and the incidence of adverse reactions within 7 days after each dose vaccination. The secondary endpoints in phase 2 trial for safety were the incidence of adverse events and serious adverse events from the first dose to 30 days after the last dose. The secondary endpoints for humoral immunogenicity in phase 2 trial were the GMT of RBD-specific ELISA antibody responses at 14 days after the last dose vaccination, seroconversion rate and GMI of RBD-specific ELISA antibody responses at 14 days and 30 days after the last dose vaccination, and the GMT, seroconversion rate, and GMI of neutralising antibody responses against live virus.

### Statistical analysis

The determination of sample size was not based on statistical power calculations, but the Technical Guidelines released by the National Medical Products Administration, China, which recommend the sample size of each dose group in phase 1 trial is about 20–30 participants. The sample size for phase 2 trial was practical. Number and percentage of participants who experienced adverse reactions after vaccination were compared among groups. The SARS-CoV-2 antibodies were presented as GMT with 95% CIs. Classification data were analysed by *χ*^2^ test or Fisher’s exact test, antibody titres after log transformed were analysed by ANOVA, and non-normally distributed data were analysed by Wilcoxon rank-sum test. Pairwise comparisons were made if a significant overall difference was found among groups, and the differences were estimated with 95% CIs. All statistical analysis uses SAS 9.4 for two-sided hypothesis testing. When the p value was less than or equal to 0.05, the difference was considered to be statistically significant.

Before the initiation of the phase 1 trial, an independent data and safety monitoring committee was established. The committee evaluated and reviewed the safety data within 7 days after the first dose vaccination to guarantee that sufficient waiting time was available between dose escalation. These two studies have been registered on ClinicalTrials.gov, NCT04530656 and NCT04640402.

### Role of the funding source

The founders of the studies participated in the design of protocols, but did not participate in data collection, statistical analysis or report writing. All authors are ultimately responsible for the publication decision.

## Supplementary information

Supplementary materials

## Data Availability

We support the sharing of participants’ data after the removal of identifiers. Both clinical trials are under way, and all individual data will not be available until the end of the one-year immune persistence observation. Any request for data sharing should be sent to jszfc@vip.sina.com, and will be reviewed and licensed on the basis of scientific merit by the sponsor, investigators and all collaborators. In order to gain access, data sharing applicants need to sign the data access agreement.
