# Supplementary material for: Safety and immunogenicity of a recombinant COVID-19 vaccine (Sf9 cells) in healthy population aged 18 years or older: two single-center, randomised, double-blind, placebo-controlled, phase 1 and phase 2 trials
Source: Signal Transduct Target Ther. 2021 Jul 15;6:271. doi: 10.1038/s41392-021-00692-3 (PMC8281021; doi:10.1038/s41392-021-00692-3)
Supplement: Supplementary file 1 — Supplementary materials [file 41392_2021_692_MOESM1_ESM.doc]

Supplementary Materials for

Safety and immunogenicity of a recombinant COVID-19 vaccine (Sf9 cells) in healthy population aged 18 years or older: two single-center, randomised, double-blind, placebo-controlled, phase 1 and phase 2 trials

Fan-Yue Meng, Fan Gao, Si-Yue Jia, Xiang-Hong Wu, Jing-Xin Li, Xi-Ling Guo, Jia-Lu Zhang, Bo-Pei Cui, Zhi-Ming Wu, Ming-Wei Wei, Zhi-Long Ma, Hai-Lin Peng, Hong-Xing Pan, Lin Fan, Jing Zhang, Jiu-Qin Wan, Zhong-Kui Zhu, Xue-Wen Wang, Feng-Cai Zhu

Correspondence to: jszfc@vip.sina.com

**This PDF file includes:**

Tables S1 to S4

## Table S1: Abnormal changes in laboratory measurements after vaccination compared with baseline in phase 1 trial

| **Laboratory measures** | **Low dose**  **(0, 28 days n=24)** | **High dose**  **(0, 28 days n=24)** | **High dose**  **(0, 14, 28 days n=24)** | **Placebo**  **(n=24)** | **p value** |
| --- | --- | --- | --- | --- | --- |
| **Adult group** |  |  |  |  |  |
| ***3 days after the first dose*** |  |  |  |  |  |
| Lymphocyte decrease | 0 (0%) | 0 (0%) | 1 (4%) | 1 (4%) | >0.99 |
| Neutrophil decrease | 0 (0%) | 1 (4%) | 2 (8%) | 1 (4%) | 0.90 |
| Platelet decrease | 0 (0%) | 0 (0%) | 1 (4%) | 0 (0%) | >0.99 |
| Hemoglobin decrease | 0 (0%) | 1 (4%) | 3 (13%) | 0 (0%) | 0.19 |
| ALT increase | 2 (8%) | 1 (4%) | 1 (4%) | 0 (0%) | 0.90 |
| AST increase | 0 (0%) | 1 (4%) | 1 (4%) | 0 (0%) | >0.99 |
| Total bilirubin increase | 2 (8%) | 0 (0%) | 0 (0%) | 1 (4%) | 0.61 |
| Blood sugar increase | 1 (4%) | 0 (0%) | 0 (0%) | 1 (4%) | >0.99 |
| Creatinine increase | 0 (0%) | 1 (4%) | 0 (0%) | 0 (0%) | >0.99 |
| PT extension | 0 (0%) | 0 (0%) | 0 (0%) | 1 (4%) | >0.99 |
| APTT extension | 2 (8%) | 0 (0%) | 3 (13%) | 0 (0%) | 0.16 |
| Urine protein increase | 2 (8%) | 4 (17%) | 1 (4%) | 3 (13%) | 0.68 |
| Urine erythrocyte increase | 1 (4%) | 1 (4%) | 1 (4%) | 2 (8%) | >0.99 |
| ***3 days after the second dose*** |  |  |  |  |  |
| WBC increase | 1 (4%) | 1 (4%) | 1 (4%) | 0 (0%) | >0.99 |
| Lymphocyte decrease | 2 (8%) | 2 (8%) | 1 (4%) | 1 (4%) | >0.99 |
| Neutrophil decrease | 0 (0%) | 2 (8%) | 1 (4%) | 2 (8%) | 0.75 |
| Platelet decrease | 2 (8%) | 0 (0%) | 0 (0%) | 0 (0%) | 0.24 |
| Hemoglobin decrease | 1 (4%) | 2 (8%) | 1 (4%) | 0 (0%) | 0.90 |
| ALT increase | 1 (4%) | 1 (4%) | 0 (0%) | 0 (0%) | >0.99 |
| AST increase | 1 (4%) | 1 (4%) | 0 (0%) | 0 (0%) | >0.99 |
| Total bilirubin increase | 1 (4%) | 0 (0%) | 1 (4%) | 0 (0%) | >0.99 |
| Blood sugar increase | 1 (4%) | 0 (0%) | 0 (0%) | 0 (0%) | >0.99 |
| Creatinine increase | 0 (0%) | 1 (4%) | 0 (0%) | 0 (0%) | >0.99 |
| PT extension | 0 (0%) | 0 (0%) | 1 (4%) | 1 (4%) | >0.99 |
| APTT extension | 1 (4%) | 0 (0%) | 4 (17%) | 1 (4%) | 0.16 |
| Urine protein increase | 1 (4%) | 3 (13%) | 2 (8%) | 1 (4%) | 0.83 |
| Urine erythrocyte increase | 0 (0%) | 7 (29%) | 2 (8%) | 3 (13%) | 0.02 |
| ***3 days after the third dose*** |  |  |  |  |  |
| Neutrophil decrease | - | - | 3 (13%) | - | - |
| Blood sugar increase | - | - | 2 (8%) | - | - |
| APTT extension | - | - | 2 (8%) | - | - |
| **Elderly group** |  |  |  |  |  |
| ***3 days after the first dose*** |  |  |  |  |  |
| WBC increase | 0 (0%) | 1 (6%) | 0 (0%) | 1 (6%) | >0.99 |
| Lymphocyte decrease | 0 (0%) | 1 (6%) | 0 (0%) | 1 (6%) | >0.99 |
| Neutrophil decrease | 0 (0%) | 1 (6%) | 0 (0%) | 0 (0%) | >0.99 |
| Platelet decrease | 0 (0%) | 0 (0%) | 1 (6%) | 3 (17%) | 0.18 |
| Hemoglobin decrease | 0 (0%) | 1 (6%) | 1 (6%) | 1 (6%) | >0.99 |
| AST increase | 0 (0%) | 0 (0%) | 2 (11%) | 0 (0%) | 0.24 |
| Total bilirubin increase | 1 (6%) | 1 (6%) | 0 (0%) | 2 (11%) | 0.90 |
| Blood sugar increase | 0 (0%) | 1 (6%) | 1 (6%) | 0 (0%) | >0.99 |
| Creatinine increase | 0 (0%) | 0 (0%) | 0 (0%) | 1 (6%) | >0.99 |
| PT extension | 0 (0%) | 1 (6%) | 0 (0%) | 0 (0%) | >0.99 |
| APTT extension | 2 (11%) | 0 (0%) | 0 (0%) | 1 (6%) | 0.61 |
| Urine protein increase | 3 (17%) | 0 (0%) | 0 (0%) | 0 (0%) | 0.06 |
| Urine erythrocyte increase | 0 (0%) | 0 (0%) | 0 (0%) | 1 (6%) | >0.99 |
| ***3 days after the second dose*** |  |  |  |  |  |
| WBC increase | 1 (6%) | 0 (0%) | 0 (0%) | 3 (17%) | 0.18 |
| Lymphocyte decrease | 1 (6%) | 0 (0%) | 1 (6%) | 3 (17%) | 0.38 |
| Neutrophil decrease | 1 (6%) | 1 (6%) | 0 (0%) | 1 (6%) | >0.99 |
| Platelet decrease | 1 (6%) | 1 (6%) | 0 (0%) | 1 (6%) | >0.99 |
| Hemoglobin decrease | 1 (6%) | 0 (0%) | 0 (0%) | 1 (6%) | >0.99 |
| ALT increase | 0 (0%) | 0 (0%) | 0 (0%) | 1 (6%) | >0.99 |
| AST increase | 1 (6%) | 3 (17%) | 1 (6%) | 1 (6%) | 0.71 |
| Total bilirubin increase | 0 (0%) | 0 (0%) | 1 (6%) | 2 (11%) | 0.61 |
| Blood sugar increase | 1 (6%) | 2 (11%) | 1 (6%) | 0 (0%) | 0.90 |
| Creatinine increase | 0 (0%) | 0 (0%) | 0 (0%) | 1 (6%) | >0.99 |
| APTT extension | 0 (0%) | 2 (11%) | 0 (0%) | 0 (0%) | 0.24 |
| Urine protein increase | 1 (6%) | 1 (6%) | 1 (6%) | 0 (0%) | >0.99 |
| ***3 days after the third dose*** |  |  |  |  |  |
| Hemoglobin decrease | - | - | 2 (11%) | - | - |
| AST increase | - | - | 2 (11%) | - | - |
| Total bilirubin increase | - | - | 1 (6%) | - | - |
| Blood sugar increase | - | - | 1 (6%) | - | - |

Data are n (%). ALT= alanine aminotransferase. AST= aspartate aminotransferase. PT=prothrombin time. APTT=activated partial thromboplastin time. WBC=white blood cell.

## Table S2: Unsolicited adverse reactions within 28 days after vaccination in phase 1 trial

|  | | **Adult group** | | | | |  | **Elderly group** | | | | |
| --- | --- | --- | --- | --- | --- | --- | --- | --- | --- | --- | --- | --- |
|  | | Low dose  (0, 28 days n=24) | High dose  (0, 28 days n=24) | High dose  (0, 14, 28 days n=24) | Placebo  (n=24) | p value |  | Low dose  (0, 28 days n=18) | High dose  (0, 28 days n=18) | High dose  (0, 14, 28 days n=18) | Placebo  (n=18) | p value |
|  | Any | 1 (4%) | 3 (13%) | 3 (13%) | 2 (8%) | 0.87 |  | 0 (0%) | 1 (6%) | 0 (0%) | 3 (17%) | 0.18 |
|  | Muscle pain | 0 (0%) | 0 (0%) | 0 (0%) | 1 (4%) | >0.99 |  | - | - | - | - | - |
|  | Throat pain | 0 (0%) | 0 (0%) | 1 (4%) | 0 (0%) | >0.99 |  | - | - | - | - | - |
|  | Dizzy | 0 (0%) | 0 (0%) | 1 (4%) | 0 (0%) | >0.99 |  | 0 (0%) | 0 (0%) | 0 (0%) | 1 (6%) | >0.99 |
|  | Eyelid itchy | 0 (0%) | 0 (0%) | 1 (4%) | 0 (0%) | >0.99 |  | - | - | - | - | - |
|  | Injection site discomfort | 0 (0%) | 2 (8%) | 0 (0%) | 1 (4%) | 0.61 |  | - | - | - | - | - |
|  | Injection site pain | 1 (4%) | 1 (4%) | 0 (0%) | 0 (0%) | >0.99 |  | - | - | - | - | - |
|  | pruritus | 0 (0%) | 0 (0%) | 1 (4%) | 0 (0%) | >0.99 |  | - | - | - | - | - |
|  | Throat discomfort | - | - | - | - | - |  | 0 (0%) | 0 (0%) | 0 (0%) | 1 (6%) | >0.99 |
|  | Sleepy | - | - | - | - | - |  | 0 (0%) | 1 (6%) | 0 (0%) | 0 (0%) | >0.99 |
|  | Head discomfort | - | - | - | - | - |  | 0 (0%) | 0 (0%) | 0 (0%) | 1 (6%) | >0.99 |

Data are n (%). Any refers to all the participants with any grade adverse reactions. Adverse reactions were graded according to the scale issued by the China State Food and Drug Administration.

## Table S3: Unsolicited adverse reactions within 30 days after vaccination in phase 2 trial

|  | | **Adult group** | | | | | |  | **Elderly group** | | | | | |
| --- | --- | --- | --- | --- | --- | --- | --- | --- | --- | --- | --- | --- | --- | --- |
|  | | Low dose  (0, 21 days n=100) | High dose  (0, 21 days n=100) | Low dose  (0, 14, 28 days n=100) | High dose  (0, 14, 28 days n=100) | Placebo  (n=80) | p value |  | Low dose  (0, 21 days n=100) | High dose  (0, 21 days n=100) | Low dose  (0, 14, 28 days n=100) | High dose  (0, 14, 28 days n=99) | Placebo  (n=80) | p value |
|  | Any | 2 (2%) | 6 (6%) | 0 (0%) | 6 (6%) | 2 (3%) | 0.05 |  | 1 (1%) | 2 (2%) | 1 (1%) | 1 (1%) | 4 (5%) | 0.37 |
|  | Stomachache | 1 (1%) | 1 (1%) | 0 (0%) | 0 (0%) | 0 (0%) | >0.99 |  | 0 (0%) | 0 (0%) | 1 (1%) | 0 (0%) | 0 (0%) | >0.99 |
|  | Dry eye | 0 (0%) | 0 (0%) | 0 (0%) | 1 (1%) | 0 (0%) | >0.99 |  | - | - | - | - | - | - |
|  | Joint pain | 0 (0%) | 0 (0%) | 0 (0%) | 1 (1%) | 0 (0%) | >0.99 |  | 1 (1%) | 0 (0%) | 1 (1%) | 0 (0%) | 0 (0%) | >0.99 |
|  | Throat discomfort | 0 (0%) | 0 (0%) | 0 (0%) | 1 (1%) | 0 (0%) | >0.99 |  | - | - | - | - | - | - |
|  | Cough | 1 (1%) | 0 (0%) | 0 (0%) | 0 (0%) | 0 (0%) | >0.99 |  | 0 (0%) | 1 (1%) | 0 (0%) | 0 (0%) | 2 (3%) | 0.11 |
|  | Insomnia | 0 (0%) | 0 (0%) | 0 (0%) | 1 (1%) | 0 (0%) | >0.99 |  | - | - | - | - | - | - |
|  | Headache | 0 (0%) | 0 (0%) | 0 (0%) | 1 (1%) | 0 (0%) | >0.99 |  | - | - | - | - | - | - |
|  | Dizzy | 0 (0%) | 1 (1%) | 0 (0%) | 1 (1%) | 1 (1%) | 0.78 |  | 0 (0%) | 1 (1%) | 0 (0%) | 0 (0%) | 0 (0%) | >0.99 |
|  | Throat irritation | 0 (0%) | 0 (0%) | 0 (0%) | 1 (1%) | 0 (0%) | >0.99 |  | - | - | - | - | - | - |
|  | Injection site discomfort | 0 (0%) | 4 (4%) | 0 (0%) | 0 (0%) | 2 (3%) | 0.01 |  | 0 (0%) | 0 (0%) | 0 (0%) | 0 (0%) | 1 (1%) | 0.17 |
|  | Dry throat | - | - | - | - | - | - |  | 0 (0%) | 0 (0%) | 0 (0%) | 0 (0%) | 1 (1%) | 0.17 |

Data are n (%). Any refers to all the participants with any grade adverse reactions. Adverse reactions were graded according to the scale issued by the China State Food and Drug Administration.

## Table S4: List of severe (grade 3) adverse reactions reported within 30 days of follow-up in phase 2 trial.

| **Dose group** | **Symptom** | **Grade** | **Start time** | **Resolve time** | **Treatment** |
| --- | --- | --- | --- | --- | --- |
| High dose (0, 14, 28 days) | Redness | 3 | After the third dose vaccination | 72h | No treatment |
| High dose (0, 14, 28 days) | Swelling | 3 | After the third dose vaccination | 72h | No treatment |
